# Blind Deblurring Method for CASEarth Multispectral Images Based on Inter-Band Gradient Similarity Prior

**DOI:** 10.3390/s24196259

**Published:** 2024-09-27

**Authors:** Mengying Zhu, Jiayin Liu, Feng Wang

**Affiliations:** 1 Aerospace Information Research Institute, Chinese Academy of Sciences, Beijing 100094, China; zhumengying221@mails.ucas.ac.cn (M.Z.); wangfeng003020@aircas.ac.cn (F.W.); 2 Key Laboratory of Technology in Geo-Spatial Information Processing and Application System, Chinese Academy of Sciences, Beijing 100190, China; 3 School of Electronic, Electrical and Communication Engineering, University of Chinese Academy of Sciences, Beijing 101408, China; 4 Key Laboratory of Target Cognition and Application Technology (TCAT), Chinese Academy of Sciences, Beijing 100190, China

**Keywords:** inter-band gradient similarity prior, blind deblurring, CASEarth, multispectral image

## Abstract

Multispectral remote sensing images contain abundant information about the distribution and reflectance of ground objects, playing a crucial role in target detection, environmental monitoring, and resource exploration. However, due to the complexity of the imaging process in multispectral remote sensing, image blur is inevitable, and the blur kernel is typically unknown. In recent years, many researchers have focused on blind image deblurring, but most of these methods are based on single-band images. When applied to CASEarth satellite multispectral images, the spectral correlation is unutilized. To address this limitation, this paper proposes a novel approach that leverages the characteristics of multispectral data more effectively. We introduce an inter-band gradient similarity prior and incorporate it into the patch-wise minimal pixel (PMP)-based deblurring model. This approach aims to utilize the spectral correlation across bands to improve deblurring performance. A solution algorithm is established by combining the half-quadratic splitting method with alternating minimization. Subjectively, the final experiments on CASEarth multispectral images demonstrate that the proposed method offers good visual effects while enhancing edge sharpness. Objectively, our method leads to an average improvement in point sharpness by a factor of 1.6, an increase in edge strength level by a factor of 1.17, and an enhancement in RMS contrast by a factor of 1.11.

## 1. Introduction

Satellite remote sensing is a technology that gathers information about ground objects from a long-distance imaging platform. With the advancement in space technology, remote sensing has become a crucial method for information detection. The CASEarth satellite is the world’s first scientific satellite dedicated to serving the “United Nations’ 2030 Agenda for Sustainable Development.” It is also the first earth science satellite of the Chinese Academy of Sciences, providing essential data for studying human–nature interactions [1]. During the operation of the CASEarth multispectral imager (MII), various factors, such as atmospheric turbulence [2,3], camera defocus [4], and platform jitter [5,6], can interfere with the collection of electromagnetic wave information. The combined effects of these factors result in blurring of the CASEarth satellite’s multispectral images, leading to degraded performance in subsequent applications, such as water monitoring [7], object recognition [8], and change detection [9]. Therefore, deblurring CASEarth multispectral images is essential.

Until now, many deblurring methods have been proposed. Earlier studies focused primarily on methods for single-image deblurring, which can be broadly categorized into optimization-based methods and deep learning-based methods. Optimization-based methods are based on the Maximum A Posteriori (MAP) framework, which incorporates prior information. The sparse gradient prior was first introduced into image deblurring by Fergus et al. [10]. Since then, the gradient sparsity prior has been widely utilized by incorporating various forms of gradient norms into models, including the L1 norm [11], the Lp (0 < *p* < 1) norm [12,13,14], and the L0 norm [15,16,17,18]. In addition to the sparse gradient prior, researchers have also established priors that consider changes in local extreme pixels caused by blurring, yielding good results [19,20,21,22,23]. Recently, building on these priors, some researchers have achieved better detail preservation by improving the regularization term [24] and the data fidelity term [25].

In recent years, with the advancement in computational power and the explosion of data, deep learning has rapidly developed across various fields, particularly in image processing. Deep learning has been widely applied in several image-related areas, such as shadow extraction [26], landslide extraction [27], and person searches [28]. In the field of image deblurring, many researchers have achieved significant results through end-to-end networks [29,30,31], yielding remarkable results. Gong et al. [32] introduced a fully convolutional deep neural network (FCN) that directly estimates motion flow from a blurred image. Xu et al. [33] proposed a deep convolutional neural network specifically designed to extract sharp edges from blurred images, effectively restoring edge clarity. Zhang et al. [34] enhanced the deblurring process by incorporating additional stages for denoising and detail enhancement. Additionally, some approaches utilize deep learning to establish image priors, which are then integrated into optimization-based methods, yielding promising results [35,36,37,38].

As multiband images find broader application, researchers have begun to focus on the spectral characteristics, aiming to deblur entire multiband images simultaneously. However, estimating the blur kernel for multiband images is challenging, so most existing methods are non-blind and do not include the blur kernel estimation process. These methods are primarily designed for hyperspectral images due to their high spectral resolution. So the similarity between grayscale images of different bands [38,39,40] and the sparsity of the core tensor [41,42] serve as effective priors. However, for multispectral images, the spectral resolution is lower than that of hyperspectral images, leading to significant differences in grayscale values across different bands. Consequently, methods developed for hyperspectral images cannot be directly applied to deblur multispectral images. Additional constraints, such as structural similarity [43,44] between images, need to be introduced.

Current deblurring methods applied to the blind deblurring of CASEarth satellite multispectral images face three primary challenges:

(1) Most blind deblurring algorithms are designed for single images and tend to overlook the spectral dimension when applied to multispectral image deblurring.
(2) Most multiband deblurring algorithms are based on hyperspectral images and are non-blind. However, due to the lower spectral resolution of multispectral images, these methods are not suitable for multispectral images.
(3) Deep learning-based methods often involve numerous parameters, and the training datasets for deblurring typically do not include remote sensing images. As a result, these methods may produce unstable results when applied to remote sensing images or data outside the training sets.

To resolve these issues, we propose the inter-band gradient similarity prior and incorporate this prior into the PMP-based method [20] to leverage the information between spectral bands. The contributions of our work are as follows:

(1) We found that the gradients of images across different bands exhibit high similarity, and the gradient differences between bands in clear images are sparser than in blurred ones. Therefore, the inter-band gradient similarity prior is proposed.
(2) We propose a new deblurring model based on the inter-band gradient similarity prior and the PMP-based model, and then transform the deblurring problem into a minimization problem.
(3) A new algorithm is designed by combining the half-quadratic splitting and alternating minimization methods. This algorithm demonstrates excellent deblurring performance and is less sensitive to parameter adjustments.

The structure of this paper is as follows: Section 2 introduce the PMP-based deblurring method. Section 3 discusses the CASEarth satellite multispectral images, the inter-band gradient similarity prior, and the proposed deblurring algorithm. Section 4 presents experiments and results based on CASEarth blurred multispectral images, and compares these results with other state-of-the-art methods. Section 5 examines the advantages and limitations of the proposed method. Finally, Section 6 summarizes the work presented in this paper and offers an outlook.

## 2. Single-Image Deblurring with PMP Prior

### 2.1. MAP Framework

As is analyzed earlier, image blur is caused by a variety of factors. By combining all the potential causes into a kernel, the blurring process for a single image $X^{s}$ of size N×N can be modeled as:

(1) $$Y^{s}=h^{s}*X^{s}+N^{s}$$

where $Y^{s}$ is the blurred image, $h^{s}$ is the blur kernel, ∗ represents the convolution operator, and $N^{s}$ is the additive noise.

In practical remote sensing imaging, we only have access to the blurred image and the kernel is unknown, so the above equation has an infinite number of solutions. The maximum posterior of the blur kernel and the clear image can be expressed as:

(2) $$P(h^{s},X^{s}|Y^{s})\propto P(Y^{s}|h^{s},X^{s})P(h^{s})P(X^{s})$$

Taking the negative logarithm of each term in Equation (2), the following minimization problem is studied:

(3) $$\underset{X^{s},h^{s}}{\arg\min}\Psi (h^{s}*X^{s}-Y^{s})+\mu \phi (X^{s})+\gamma \varphi (h^{s})$$

In Equation (3), Ψ(⋅) is a data fidelity term that ensures the similarity between the clear image and the original blurred image while removing noise simultaneously. The latter two terms are called regularization terms, which are determined by prior information. ϕ(⋅) represents regularization term for the clear image, while φ(⋅) is a kernel regularization term to smooth the kernel. The number of regularization terms for the clear image and blur kernel can vary depending on the problem’s specific characteristics. However, more regularization terms also mean increased computational complexity. μ and γ are positive weighting parameters used to balance the data fidelity term and each regularization term.

Using the alternating minimization algorithm, the model in Equation (3) can be split into two sub-problems:

(4) $$\underset{X^{s}}{\arg\min}\Psi (h^{s}*X^{s}-Y^{s})+\mu \phi (X^{s})$$

(5) $$\underset{h^{s}}{\arg\min}\Psi (h^{s}*X^{s}-Y^{s})+\gamma \varphi (h^{s})$$

### 2.2. PMP-Based Deblurring Model

Wen et al. [20] proposed the PMP prior, which has been successfully applied by [45,46] to achieve good results on single-band remote sensing images. PMP is a collection of local minimal pixels over non-overlapping patches. Given a grayscale image I of size m×n, the PMP with a patch size of r×r is defined as:

(6) $$P(I)(i)={min}_{(x,y)\in \Omega_{i}}(I(x,y))$$

i=1,2,⋯P, $P=\left\lceil \frac{m}{r}\right\rceil \cdot \left\lceil \frac{n}{r}\right\rceil$, $\left\lceil \cdot \right\rceil$ represents the floor function, and $\Omega_{i}$ denotes the index set of the pixel locations for the i-th patch. (x,y) represents the pixel location.

The PMP prior is derived from the DCP prior [19], which is defined as follows:

(7) $$D(I)\left(i^{\prime }\right)=\min_{\left(x^{\prime },y^{\prime }\right)\in N_{i}}\left(I\left(x^{\prime },y^{\prime }\right)\right)$$

i=1,2,⋯mn, $N_{i}$ denotes the index set of the pixel locations of the i-th patch. $\left(x^{\prime },y^{\prime }\right)$ represents the pixel location.

For the same patch size P(I), is a subset of D(I) with less data, requiring less time to find the local minimum pixels, thereby simplifying the algorithm.

From Equation (1), it can be concluded that the grayscale value at a point after blurring can be considered a weighted sum of the grayscale value at the point and within its neighborhood. Therefore, for the pixel with the minimum grayscale value in a patch, the grayscale value after blurring will be greater than or equal to the original value. The authors analyzed the intensity histograms of over 5000 natural images for patch-wise minimal pixels of clear and blurred images, and the results show that the PMP of clear images is much sparser than that of blurred images. Subsequently, Liao et al. [47] theoretically proved that blurring will lessen the sparsity of PMP.

By using the L0 norm, the PMP-based deblurring model is expressed as:

(8) $$\underset{X}{\min}{∥X^{s}*h^{s}-Y^{s}∥}_{2}^{2}+\mu {∥\nabla X^{s}∥}_{0}+\rho {∥P\left(X^{s}\right)∥}_{0}$$

(9) $$\underset{h^{s}}{\min}{\left\|X^{s}*h^{s}-Y^{s}\right\|}_{2}^{2}+\gamma {\left\|h^{s}\right\|}_{2}^{2}$$

ρ is a positive weight for the corresponding regularization terms. The PMP-based method utilizes the gradient sparsity prior to get sharper edges. $\nabla X^{s}={\left(\nabla_{h}X^{s},\nabla_{v}X^{s}\right)}^{T}$ denotes the image gradient. By incorporating the L2 norm, this model ensures the smoothness of the blur kernel and suppresses noise.

The model has been successfully applied to deblur single images with good results. However, for the CASEarth multispectral images studied in this paper, applying this model in a band-wise manner ignores the spectral correlation. Therefore, in this paper, we extend the PMP-based model to multiband and introduce spectral correlation to enhance performance.

## 3. Multispectral Image Deblurring with Inter-Band Similarity and PMP Prior

### 3.1. CASEarth Multispectral Images

As shown in Table 1, CASEarth satellite MII has seven bands. The two deep blue bands are suitable for coastal zone and offshore environment detection; the red edge band is used for monitoring the vegetation growth; the red band, in conjunction with the red edge band, is effective for detecting suspended sediment; and the green band and near infrared band are utilized for extracting on-land vegetation coverage [1]. The band settings facilitate the observation of resource distribution.

**Figure 1:**
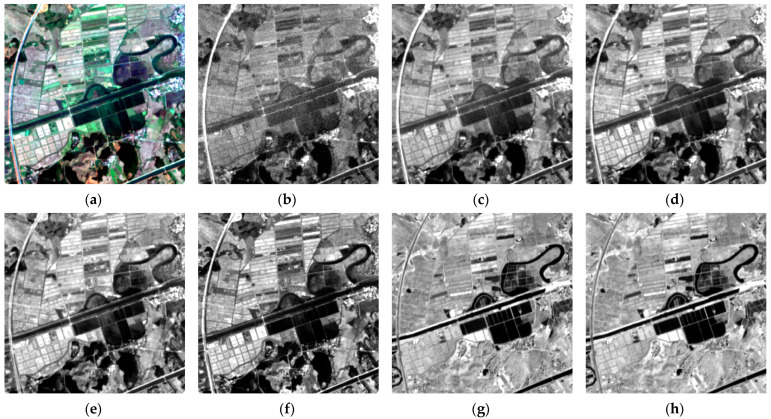
An image from the state of Florida (size: 400 × 400, stretching the grayscale values to the range of [0, 1] for better visualization): (**a**) true color image; (**b**) Band1 image; (**c**) Band2 image; (**d**) Band3 image; (**e**) Band4 image; (**f**) Band5 image; (**g**) Band6 image; (**h**) Band7 image.

From Table 1, it is evident that the signal-to-noise ratio of the B1 band is lower than that of other bands. As shown in Figure 1, the B1 band image exhibits significant noise in addition to blurring.

**Figure 2:**
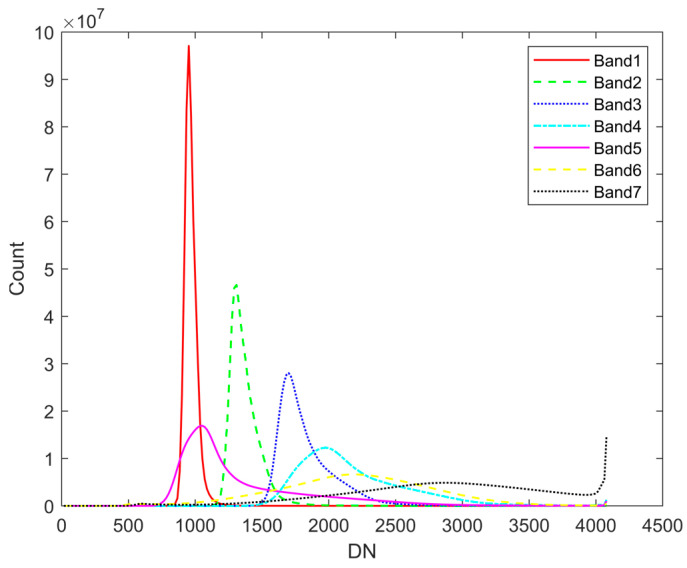
The histogram of grayscale values for seven spectral bands of one scene. DN means digital number, which refers to the grayscale value.

We analyze the histogram distribution curves of grayscale values for the seven bands of the MII one-scene image, which includes features such as mountains, vegetation, and water. As depicted in Figure 2, the overall grayscale values of B1 band are lower than other bands. This is because most features, such as vegetation, water, buildings, soils, and mountains, have low reflectance for the electromagnetic wave of the B1 band, making it more susceptible to noise, as shown in Figure 1b

### 3.2. Inter-Band Gradient Similarity Prior

The CASEarth multispectral images are positionally aligned, so they capture the same geographic area. Different grayscale values correspond to the ability of various features to reflect specific electromagnetic waves, resulting in variations in grayscale values across different bands. However, the gradient of the image reveals the distribution of features. Edges with a larger gradient typically mark the boundaries between different types of features. Since the distribution of features is fixed during imaging, the gradient differences between different bands should be sparse.

The human eye is less sensitive to grayscale images than to color images, so we present Figure 1d–f along with their corresponding gradient image as heatmaps.

**Figure 3:**
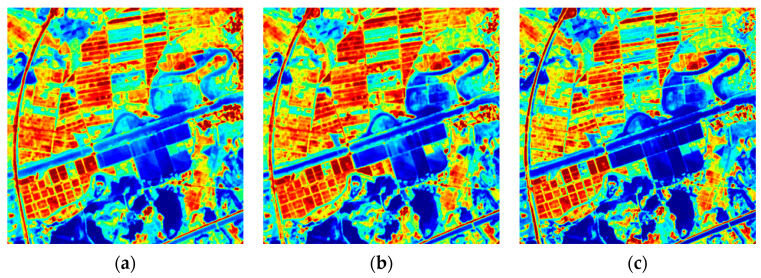

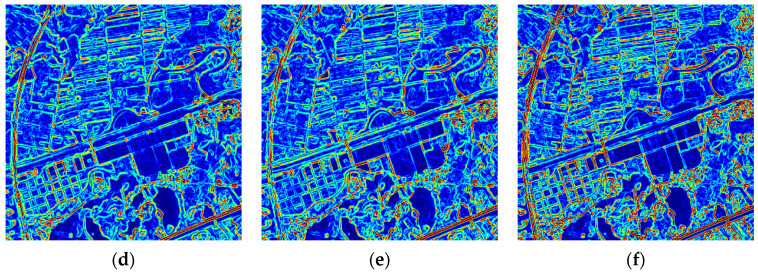
Heatmaps of Figure 1d–f and their gradient images: (**a**) Band3 image; (**b**) Band4 image; (**c**) Band5 image; (**d**) Band3 gradient image; (**e**) Band4 gradient image; (**f**) Band5 gradient image.

Figure 3 demonstrates that while the grayscale images display varying values in many regions, the gradient images differ less from each other, and thus the gradient images are more similar.

Fergus et al. [10] point out that the gradient distribution in clear images obeys a heavy-tail distribution, which means that most gradient values in clear images are close to zero, and that blur makes the gradient of an image less sparse. This is easy to understand because blur smooths out the gradients at the edges and increases edge width.

**Figure 4:**
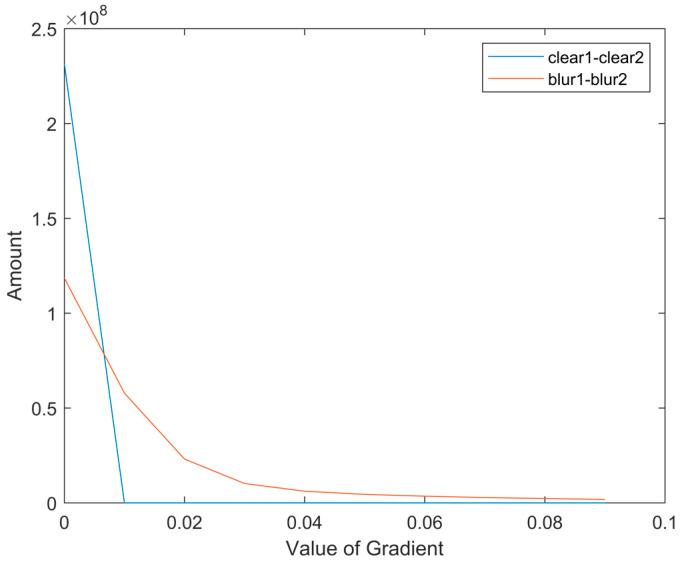
The histogram of absolute gradient difference values for a single-scene image.

To test whether blur reduces the sparsity of gradient differences between bands, we selected a single-scene multispectral image from the Sentinel-2 satellite, with a single band size of 10,980 × 10,980 pixels. We applied the real kernel estimated by our method, which will be described later in Section 3.3, and calculated the absolute value of gradient differences between the green and blue bands. Figure 4 illustrates that smaller values of gradient differences are more likely to occur when the image is clear. In the clear image, the differences in the gradient are closer to 0 and the variance is smaller. This demonstrates that the gradient difference between clear bands exhibits higher sparsity. Therefore, the proposed prior is more conductive to clear images, which proves the effectiveness of the proposed prior.

### 3.3. Deblurring Algorithm with Inter-Band Similarity Prior and PMP Prior

Denote $X^{l}$ as the image of the l-th band of multispectral image X, whose size is N×N×L. We can rewrite Equation (1) as:

(10) $$Y^{l}=h^{l}*X^{l}+N^{l}$$

$Y^{l}$, $h^{l}$, and $N^{l}$ represent the blurred image, blur kernel, and noise of the l-th band, respectively. By stacking the elements of the matrix row-wise in lexicographic order to form a vector, the convolution can be expressed as a matrix–vector product form, that is,

(11) $$y^{l}=H^{l}x^{l}+n^{l}$$

$y^{l}$, $x^{l}$, and $n^{l}$ are the vector forms of $Y^{l}$, $X^{l}$, and $N^{l}$, respectively. $H^{l}$ can be obtained from $h^{l}$ by the convolution formula. The specific expression for $H^{l}$ is as follows:

(12) $$H^{l}=\left[\begin{array}{cccc} H_{1}^{l} & H_{N}^{l} & \cdots & H_{2}^{l}\\ H_{2}^{l} & H_{1}^{l} & \cdots & H_{3}^{l}\\ \vdots & \vdots & \ddots & \vdots \\ H_{N}^{l} & H_{N-1}^{l} & \cdots & H_{1}^{l} \end{array}\right],\;H_{i}^{l}=\left[\begin{array}{cccc} h_{i1}^{l} & h_{iN}^{l} & \cdots & h_{i2}^{l}\\ h_{i2}^{l} & h_{i1}^{l} & \cdots & h_{i3}^{l}\\ \vdots & \vdots & \ddots & \vdots \\ h_{iM}^{l} & h_{iM-1}^{l} & \cdots & h_{i1}^{l} \end{array}\right]$$

$H_{i}^{l}\in R^{N\times N}$ is a circulant matrix, and $H^{l}\in R^{N^{2}\times N^{2}}$ is a block–circulant matrix.

For a multispectral image with L bands, we can stack all bands into a vector $x={\left[x^{1},x^{2},\cdots x^{L}\right]}^{T}$; then, the multiband deblurring model can be expressed as:

(13) y=Hx+n

where $x,y,n\in R^{N^{2}L}$. The matrix $H\in R^{N^{2}L\times N^{2}L}$ contains the blur kernel information of all bands.

With the multispectral blur model, combing the PMP-based method [18] and the proposed inter-band gradient similarity prior, we establish our model for latent image estimation:

(14) $$\underset{x}{\min}{\left\|Hx-y\right\|}_{2}^{2}+\mu {\left\|Dx\right\|}_{0}+\rho {\left\|P(x)\right\|}_{0}+\lambda {\left\|D_{b}Dx\right\|}_{1}$$

λ is a positive parameter that adjusts the degree of regularization. D and $D_{b}$ are the convolution matrix for the spatial and spectral finite-difference operator, respectively. $D_{b}Dx$ represents the inter-band gradient difference, and we use the L1 norm to impose the sparse constraint. It should be noted that we do not need to know the exact form of the matrices D and $D_{b}$, because the final solution of the model is computed by 3D-DFT.

For the kernel estimation, it is challenging to define the prior of H, which comprises blur kernels of all bands. Considering that each band of CASEarth satellite multispectral images is coupled together, and the electromagnetic waves of different wavelengths are projected onto the corresponding CMOS slices through the reflector, we assume that the kernel of each band is the same. In the stage of kernel estimation, noise condition affects the accuracy of the kernel estimation. Therefore, we use the signal-to-noise (SNR) parameter to select the single-band image Y with the lowest noise level for kernel estimation. We rewrite Equation (9) as:

(15) $$\underset{h}{\min}{\left\|X*h-Y\right\|}_{2}^{2}+\gamma {\left\|h\right\|}_{2}^{2}$$

The following part of Section 3 describes the solving algorithms for estimating latent clear image, kernel estimation, and an additional denoising step for the B1 band.

#### 3.3.1. Latent Image Estimation

Since the L0 norm is nonconvex, solving it directly is challenging. For the PMP term, we use a thresholding/shrinkage step in the iterative procedure to ensure the PMP value is nonincreasing. The specific steps are as follows:

In the whole optimization process, we employ a two-layer approach consisting of an inner and outer loop. The outer loop iteratively estimates the latent clear image and kernel, and the inner loop imposes constraints. Assume the current iteration is the m-th outer loop and the j-th inner loop. For the resulting image from the previous inner loop iteration $x^{m,j-1}$, we find its patch-wise minimal pixel set. Let $\Pi^{m,j-1}$ record the position of PMP pixels in the image, and the mask matrix $M^{m,j-1}$ can be described as:

(16) $$M^{m,j-1}(x,y)=\left\{\begin{array}{ll} 1, & (x,y)\in \Pi^{m,j-1}\\ 0, & otherwise \end{array}\right.$$

Then, we update the latent image as:

(17) $${\tilde{x}}^{m,j-1}=x^{m,j-1}\circ (1-M^{m,j-1})+\mathrm{Thr}(x^{m,j-1}\circ M^{m,j-1},\tau )$$

∘ is the elementwise multiplication. The function Thr(⋅) zeroes out the intensity of elements below threshold τ while leaving the intensity of other elements unchanged. Through iterative steps, the sparsity of the estimated latent image is improved. Function Thr(⋅) is defined as follows:

(18) $$\mathrm{Thr}(Ζ,\tau )(x,y)=\left\{\begin{array}{ll} 0, & |Ζ(x,y)|<\tau \\ Ζ(x,y), & otherwise \end{array}\right.$$

From Equation (18), we can see that only patch-wise minimal pixels whose intensity is below the threshold are updated. After imposing the PMP constraints, the model becomes:

(19) $$\underset{x}{\min}{\left\|Hx-y\right\|}_{2}^{2}+\mu {\left\|Dx\right\|}_{0}+\lambda {\left\|D_{b}Dx\right\|}_{1}$$

The above model can be solved through the half quadratic splitting algorithm by introducing auxiliary variables w and z:

(20) $$\underset{x}{\min}{\left\|Hx-y\right\|}_{2}^{2}+\mu {\left\|w\right\|}_{0}+\lambda {\left\|z\right\|}_{1}+\alpha {\left\|Dx-w\right\|}_{2}^{2}+\beta {\left\|D_{b}Dx-z\right\|}_{2}^{2}$$

α and β are positive penalty parameters. When α→∞ and β→∞, the solution of the optimization problem in Equation (20) is close to the solution of the original Formula (19). Equation (20) can be solved by alternating minimization, which divides it into three subproblems:

1. Estimating w

The subproblem of w is:

(21) $$\underset{w}{\min}\mu {\left\|w\right\|}_{0}+\alpha {\left\|D{\tilde{x}}^{m,j-1}-w\right\|}_{2}^{2}$$

There are similar solution problems in [15,47], and based on these papers, we can solve the above equation as:

(22) $$w^{m,j}=\left\{\begin{array}{ll} 0 & D{\tilde{x}}^{m,j-1}<\sqrt{\mu /\alpha }\\ D{\tilde{x}}^{m,j-1} & otherwise \end{array}\right.$$

2. Estimating z

The subproblem of z is:

(23) $$\underset{z}{\min}\lambda {\left\|z\right\|}_{1}+\beta {\left\|D_{b}D{\tilde{x}}^{m,j-1}-z\right\|}_{2}^{2}$$

It can be solved by using soft thresholding [48]:

(24) $$z^{m,j}=\mathrm{sign}(D_{b}D{\tilde{x}}^{m,j-1})\circ \max(D_{b}D{\tilde{x}}^{m,j-1}-\lambda /2\beta ,0)$$

where the function sign(⋅) retains the sign of the input data.

3. Estimating x

(25) $$\underset{x}{\min}{\left\|Hx-y\right\|}_{2}^{2}+\alpha {\left\|Dx-w^{m,j}\right\|}_{2}^{2}+\beta {\left\|D_{b}Dx-z^{m,j}\right\|}_{2}^{2}$$

It is a simple least-squares problem, and we can solve it by setting the partial derivative to zero, yielding the following linear equation:

(26) $$(H^{T}H+\alpha D^{T}D+\beta D^{T}D_{b}^{T}D_{b}D)x=H^{T}y+\alpha D^{T}w^{m,j}+\beta D^{T}D_{b}^{T}z^{m,j}$$

For multiband images, the size of the matrix H is $N^{2}L\times N^{2}L$, and the kernel will occupy a large memory space, so we solve the above equation by the 3D-DFT:

(27) $$x=F^{-1}\left\{\left.\frac{F(H^{T}y+\alpha D^{T}w^{m,j}+\beta D^{T}D_{b}^{T}z^{m,j})}{F(H^{T}H+\alpha D^{T}D+\beta D^{T}D_{b}^{T}D_{b}D)}\right\}\right.$$

#### 3.3.2. Blur Kernel Estimation

Equation (15) can be solved in the image gradient domain for better results:

(28) $$\underset{h}{\min}{\left\|\nabla X*h-\nabla Y\right\|}_{2}^{2}+\gamma {\left\|h\right\|}_{2}^{2}$$

Equation (28) appears to be a least-squares problem, and by 2D-DFT we can obtain:

(29) $$\underset{F(h)}{\min}{\left\|F(\nabla X)\circ F(h)-F(\nabla Y)\right\|}_{2}^{2}+\gamma {\left\|F(h)\right\|}_{2}^{2}$$

The closed form of the above equation is:

(30) $$h=F^{-1}(\frac{\bar{F(\nabla_{h}X)}\circ F(\nabla_{h}Y)+\bar{F(\nabla_{v}X)}\circ F(\nabla_{v}Y)}{\bar{F(\nabla_{h}X)}\circ F(\nabla_{h}X)+\bar{F(\nabla_{v}X)}\circ F(\nabla_{v}X)+\gamma I_{0}})$$

#### 3.3.3. B1 Band Image Denoising Method

Various deblurring methods are also committed to recovering the texture information of the image, but when there is a significant amount of noise in the image, direct deblurring can amplify the noise as if it were part of the texture. Therefore, for CASEarth multispectral B1 band images, certain denoising steps must be adopted to prevent the noise from being amplified.

Denoising is typically achieved through smoothing steps, which inevitably result in some loss of image details. Petschnigg et al. [49]. proposed a joint bilateral filtering, which sets the weights based on the spatial distance of the original image and the grayscale variations in the reference image to preserve edges. The joint bilateral filtering is as follows:

(31) $$f_{p}=\frac{1}{k(p)}\sum_{p^{\prime }\in T_{p}}g_{d}(p^{\prime }-p)g_{r}(F_{p^{\prime }}-F_{p})f_{p^{\prime }}$$

(32) $$k(p)=\sum_{p^{\prime }\in T_{p}}g_{d}(p^{\prime }-p)g_{r}(F_{p^{\prime }}-F_{p})$$

where F is the reference image, p is the center pixel of the image patch to be denoised, $T_{p}$ is the neighborhood of pixel p, and $p^{\prime }$ indicates the position of a pixel within this neighborhood. Both $g_{d}$ and $g_{r}$ are Gaussian functions, representing the spatial domain weight of the original image and the grayscale domain weight of the reference image, respectively. Their widths are determined by $\sigma_{d}$ and $\sigma_{v}$.

Joint bilateral filtering achieves edge-preserving effects by applying smaller grayscale domain weights to points with larger grayscale differences in the reference image. Therefore, the similarity between the reference image and B1 band image influences the result. For optical remote sensing images, the grayscale values of different bands reflect the ability of ground objects to reflect electromagnetic waves at specific wavelengths. Since the reflection spectrum curves of ground objects are mostly continuous, theoretically, the closer the bands are to each other, the higher the similarity between the images.

The normalized cross-correlation (NCC) coefficient can measure the similarity between images. The formula for the NCC coefficient is shown in Equation (33). Table 2 shows the NCC coefficient values between the noisy image of the B1 band and other bands in Figure 1.

(33) $$NCC=\frac{\sum_{i}\sum_{j}\left[f(i,j)-\mu_{f}][g(i,j)-\mu_{g}\right]}{\sigma_{f}\sigma_{g}}$$

$\mu_{f}$, $\mu_{g}$, $\sigma_{f}$, and $\sigma_{g}$ represent the mean and standard deviation of images f and g, respectively. The greater the consistency in their grayscale value changes, the larger the NCC coefficient, indicating higher image similarity.

As shown in the table, the similarity between the B2 band and B1 band images is the highest. This result is consistent with our theoretical analysis, so we use the B2 band data as the reference image. In addition to selecting the reference image, the values of parameters $\sigma_{d}$ and $\sigma_{v}$ are also closely related to the denoising performance.

The spatial domain parameter $\sigma_{d}$ determines the weight distribution within the spatial neighborhood—specifically how the distance between a pixel and its neighboring pixels influences the weights. A smaller spatial domain variance allows the filter to emphasize distance differences between neighboring pixels, thereby better preserving edges and details in the image. However, when the image contains significant noise, a smaller variance may result in insufficient smoothing, failing to effectively remove the noise. Conversely, a larger spatial domain variance broadens the filter’s range of influence, enhancing noise smoothing but potentially blurring image details.

The pixel domain parameter $\sigma_{v}$ controls how differences in pixel intensity values affect the weights. A smaller pixel domain variance makes the filter more sensitive to changes in the reference image’s intensity values, thereby better preserving structural information and edge details in the image.

As analyzed, these two parameters are independent of each other. Therefore, we first focus on the denoising problem. Experimental results indicate that when $\sigma_{d}$ is less than 2.5, significant noise remains in the image, while when $\sigma_{d}$ exceeds 4, there is considerable loss of detail. Next, we consider the preservation of structural information. By keeping $\sigma_{d}$ within the range of [2.5, 4], we found that when $\sigma_{v}$ is greater than 0.01, blocky regions appear in the image, indicating that only large details are preserved. However, when $\sigma_{v}$ is less than 0.005, the image appears more visually messy than other results. Therefore, this study selects parameters $\sigma_{d}$ and $\sigma_{v}$ within the ranges of [2.5, 4] and [0.005, 0.01], respectively.

**Figure 5:**
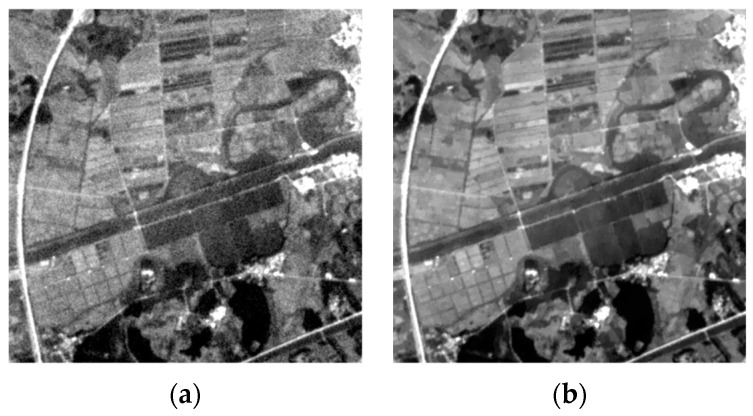
Denoising and deblurring results of B1 band images: (**a**) original image; (**b**) deblurring result.

Figure 5 illustrates the results of deblurring and denoising in the B1 band when $\sigma_{d}=3$ and $\sigma_{v}=0.005$.

**Figure 6:**
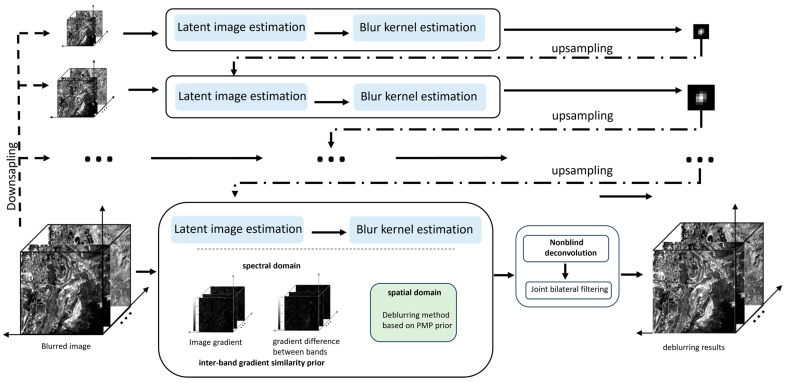
The structure flowchart.

The flowchart of the proposed deblurring method is shown as Figure 6. For blurred multispectral images of CASEarth, we use a coarse-to-fine image pyramid structure to utilize the multiscale features. At each image scale, constraints are imposed in both spectral and spatial domains according to the characteristics of multispectral images. Firstly, in the spectral domain, we compute the inter-band gradient difference matrix, and the spectral constraints are realized by the sparsity of the gradient difference matrix. In the spatial domain, the gradient sparsity constraints and the local minimum pixel intensity constraints are realized by the method based on the PMP prior. A blur kernel is obtained at each scale, and then the kernel is up-sampled to serve as the initial kernel estimation for the next scale image. At the end of the multiscale estimation algorithm, the clear image is obtained using a non-blind deconvolution algorithm [50]. In the end, for the B1 band affected by noise, the joint bilateral filtering algorithm is applied.

## 4. Experiments and Results

### 4.1. Experiment Setup and Evaluation Metrics

To verify the deblurring performance of the proposed method on real CASEarth multispectral images, we choose five other state-of-the-art blind image-deblurring algorithms, including LMG [51], NSM [52], Max–min [23], PMP [20], and CPMMP [45]. These five methods all establish image priors based on the effects of blurring. The LMG prior is based on the observation that the maximum value of a local patch gradient will diminish after the blurring process. The max–min method finds out that the difference between the highest and lowest intensities around dominant edges is greater than in smooth areas, and blurring greatly diminishes this inherent characteristic. The Normalized Sparsity Measure (NSM) prior is the ratio of the L1 norm to the L2 norm of image gradients, which can compensate for the attenuation of high frequencies. The PMP prior leverages the characteristic that the strengths of patch-wise minimum pixels in a clear image increase after blurring, achieving good results in single-band image deblurring. The CPMMP prior considers that the local maximum values of an image decrease after blurring and has demonstrated good performance on GF-2 satellite multispectral out-of-focus images.

The CASEarth multispectral images used in this paper are Level 4 images. The data format is 16 bits, but CASEarth only utilizes 12 bits, so all images are normalized to the range of [0, 1] before deblurring. The selected multispectral images used in the experiments are 400 × 400 × 7 in size. The results are presented using grayscale images for better visualization. The parameters used in the experiments were set as follows:$\mu =4\times 10^{-3}$, $\lambda =1\times 10^{-8}$, γ=2.

The metrics for evaluating image clarity can be divided into subjective and objective evaluation metrics. A good deblurring method should produce visually pleasing results. Subjective evaluation metrics refer to the human eye’s perception of image quality, but there are some details that the human eye cannot discern. Objective evaluation metrics are further divided into no-reference and reference-based metrics. In practical engineering applications, a clear reference image is often unavailable, so only objective evaluation metrics can be used to assess image quality. Therefore, to better compare the strengths and weaknesses of various methods, we select three no-reference objective evaluation metrics, including point sharpness, edge strength level, and RMS contrast [43,44], to assess the deblurring performance.

1. Point Sharpness

$$P=\frac{1}{MN}\sum_{i=1}^{MN}\sum_{a=1}^{MN}\left|\frac{df}{dx}\right|$$

f is an image of size M×N. Point sharpness is related to image gradients. It considers the variations within the pixel neighborhood and weights the pixel grayscale values in eight directions according to distance. This metric can be understood as a measure of the grayscale diffusion around a pixel. A larger value indicates a clearer image. In this paper, the neighborhood size is set to 8.

2. Edge Strength Level

$$\mathrm{ESL}=\sqrt{\frac{\sum_{edge}\nabla f_{i,j}}{N_{edge}}}$$

In the process of computing ESL, we first use the canny operator to extract the edge point set of image f. Then, as mentioned in [45], the edge area is constructed using morphological dilation of the basic edges points set with circular structuring elements of radius 3. $N_{edge}$ represents the number of points in the edge area. A higher ESL value indicates more image details.

3. RMS Contrast

$$C_{\mathrm{rms}}=\frac{\sigma }{\mu }$$

σ is the standard deviation of the image, μ is the mean value of the image, and σ reflects the extent to which the overall grayscale values of the image deviate from the mean grayscale value. Greater deviation and higher contrast mean a clearer image.

### 4.2. Experimental Results

#### 4.2.1. Deblurring Experiments and Analysis

**Figure 7:**
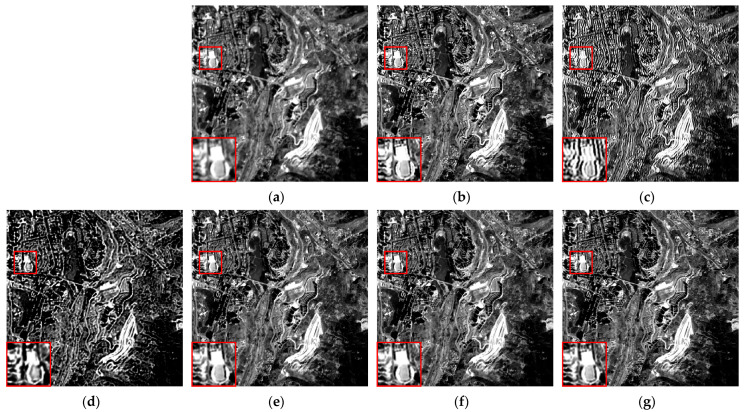
Deblurring results of MII 01: (**a**) blurred image; (**b**) LMG; (**c**) NSM; (**d**) max–min; (**e**) PMP; (**f**) CPMMP; (**g**) our result. Zoom in to view details.

**Figure 8:**
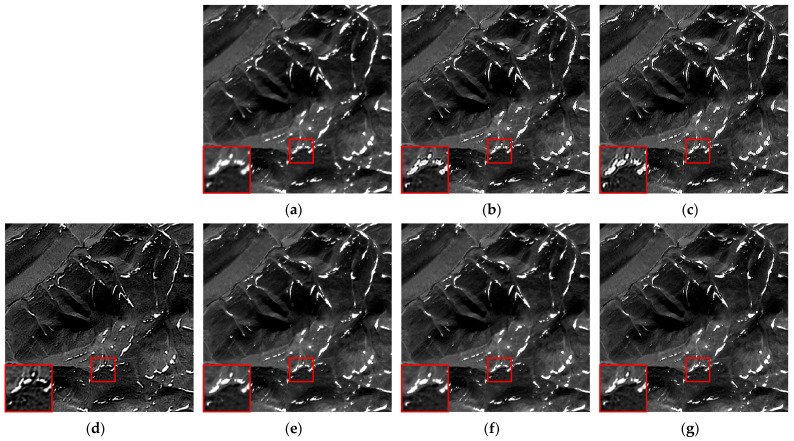
Deblurring results of MII 02: (**a**) blurred image; (**b**) LMG; (**c**) NSM; (**d**) max–min; (**e**) PMP; (**f**) CPMMP; (**g**) our result. Zoom in to view details.

**Figure 9:**
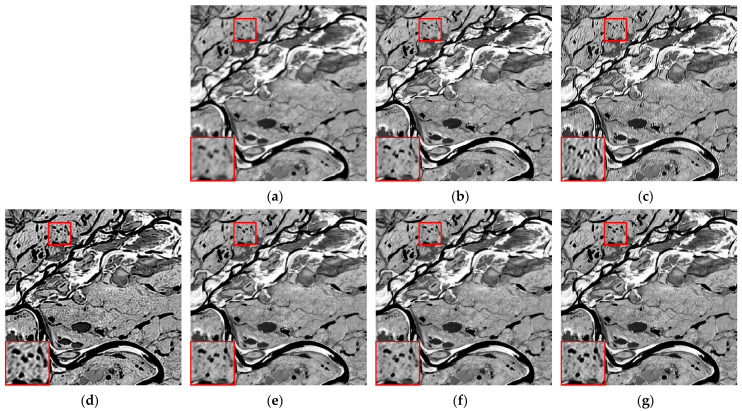
Deblurring results of MII 03: (**a**) blurred image; (**b**) LMG; (**c**) NSM; (**d**) max–min; (**e**) PMP; (**f**) CPMMP; (**g**) our result. Zoom in to view details.

**Figure 10:**
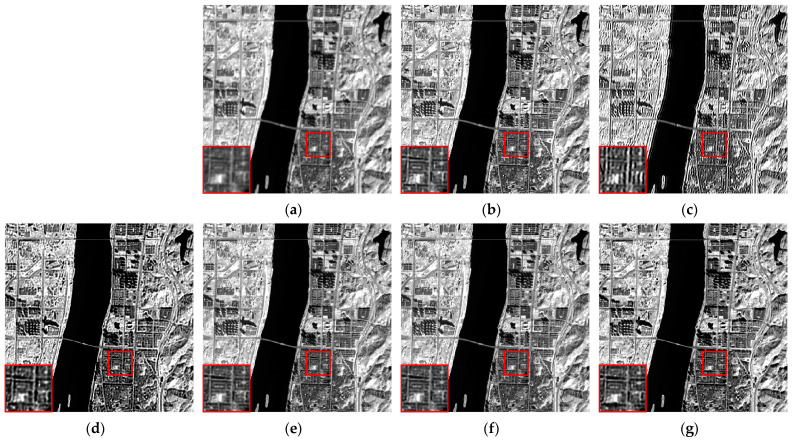
Deblurring results of MII 04: (**a**) blurred image; (**b**) LMG; (**c**) NSM; (**d**) max–min; (**e**) PMP; (**f**) CPMMP; (**g**) our result. Zoom in to view details.

**Figure 11:**
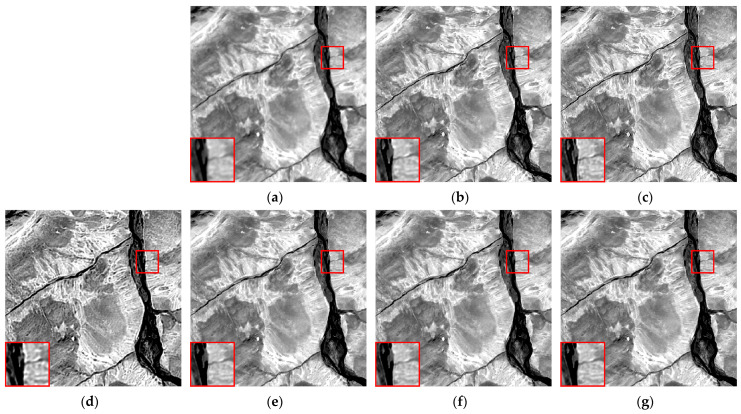
Deblurring results of MII 05: (**a**) blurred image; (**b**) LMG; (**c**) NSM; (**d**) max–min; (**e**) PMP; (**f**) CPMMP; (**g**) our result. Zoom in to view details.

**Figure 12:**
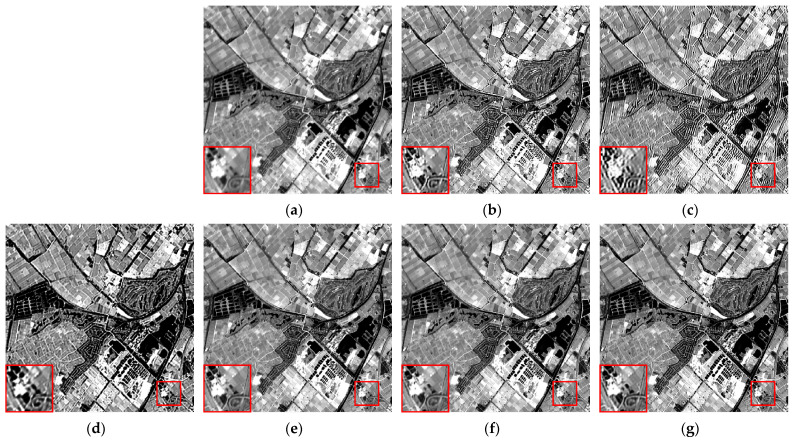
Deblurring results of MII 06: (**a**) blurred image; (**b**) LMG; (**c**) NSM; (**d**) max–min; (**e**) PMP; (**f**) CPMMP; (**g**) our result. Zoom in to view details.

We selected six multispectral images captured by the CASEarth MII, each with a spatial size of 400 × 400 pixels, and named them MII 01–MII 06. These images include samples featuring both complex and simpler textures. The experimental results are presented in Figure 7, Figure 8, Figure 9, Figure 10, Figure 11 and Figure 12.

First, we analyze the results from a subjective perspective. The PMP, CPMMP, and our proposed method deliver satisfying visual effects across all images. However, the NSM, LMG, and max–min methods perform poorly in many images, introducing artifacts that degrade visual quality. Specifically, the max–min method excessively sharpens the images, making the details appear overly messy. For the LMG method, in Figure 8b, additional image structures appear at the edges of square buildings, and in Figure 9b, which depicts mountains and snow distribution, the results show unnatural transitions at the edges of the snow. The results of NSM in Figure 7c, Figure 8c, Figure 9c, Figure 10c and Figure 12c are severely contaminated by artifacts, causing significant alterations to the image structures.

Based on the proportion of artifacts in the entire image, we can further categorize the results into two cases: severe artifacts and subtle artifacts. It is important to note that most no-reference evaluation metrics rely on the structural information of the image itself, so the metric values may vary depending on the image content. From the calculation formulas and their implications, we know that for images depicting the same content, the sharper the edges, the higher the values of the three metrics: point sharpness, edge strength level, and RMS contrast. However, images with artifacts effectively introduce additional structural information to the original image, so the more artifacts present, the higher the corresponding metric values. The over-sharpened images will also exhibit greater grayscale transitions, leading to larger metric values.

Table 3, Table 4 and Table 5 display the metric values. As discussed, images affected by artifacts and over-sharpening tend to have higher metric values. However, these values do not align with subjective visual assessments. We have used bold text to highlight the highest metric values. After removing images with artifacts and over-sharpening, we underlined the maximum values among the remaining images with good subjective visual quality. In the absence of artifacts or over-sharpening (i.e., for methods in Table 3, Table 4 and Table 5 not marked with * or ^), our method achieves the best metric results. This indicates that our method achieves the best overall results in the combined evaluation of both subjective and objective performance.

#### 4.2.2. Deblurring Performance for Each Band

**Figure 13:**
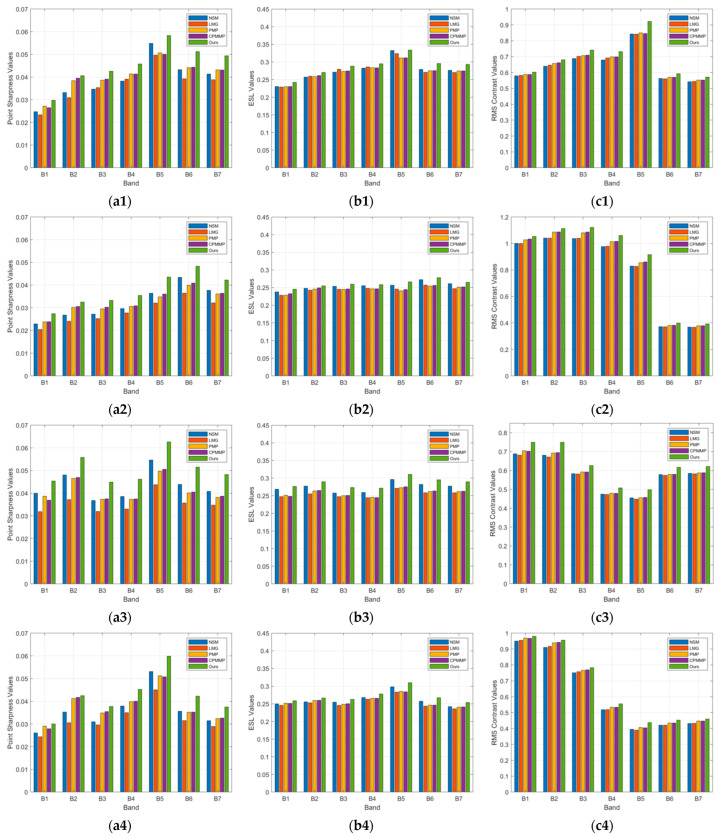
Metrics of CASEarth MII image B1–B7 bands: (**a1**) point sharpness values; (**b1**) edge strength level values; (**c1**) RMS contrast values; (**a2**) point sharpness values; (**b2**) edge strength level values; (**c2**) RMS contrast values; (**a3**) point sharpness values; (**b3**) edge strength level values; (**c3**) RMS contrast values; (**a4**) point sharpness values; (**b4**) edge strength level values; (**c4**) RMS contrast values.

In the previous section, we observe that the method proposed in this paper does not introduce artifacts. Table 3, Table 4 and Table 5 also show the average performance in metrics. We can see that artifacts affect the metric results. To analyze the improvement effect of the proposed method on each band from B1–B7, we select another four images that visually show no artifacts. However, the images produced by the max–min method exhibit significant distortion, leading to higher metric values. We have decided not to include these results here, as they do not align with the subjective evaluation. Figure 13 shows the metric changes for each band of these four images. For the B1 band, the results of each method are denoised using joint bilateral filtering, and the B2 band with the highest similarity is used as the reference image.

In Figure 13, each row corresponds to the results of three metrics for a single image. For example, in the first row, Figure 13a1–c1 display the metric values for the first image across different bands. Figure 13a1–a4 illustrate the comparison of point sharpness values across bands, Figure 13b1–b4 show the comparison of edge strength-level values, and Figure 13c1–c4 present the comparison of RMS contrast values. Figure 13 displays the metric values across bands for four artifact-free images. We can see that in artifact-free images, the metric values for all bands using our method are higher than those obtained by other methods, indicating that our method enhances edge details across all bands.

#### 4.2.3. Large-Scale Application

Remote sensing images are characterized by their wide coverage and large data volume, and typically, a single scene of a remote sensing image is relatively large in size, reaching several thousand or even tens of thousands of rows or columns. Therefore, it is essential to consider how to process large-scale data during the deblurring of remote sensing images.

Assuming that the blur kernel remains constant across the spatial domain, we can estimate the blur kernel using a small region of the image, significantly reducing the computational load. Therefore, we test the impact of image size on the proposed method, with quantitative results for the same regions of these images shown in Table 6 and Table 7. The image sizes used for estimation range from a minimum of 400 × 400 to a maximum of 4000 × 4000.

The results demonstrate that for images with a size of 400, as well as those 5 times and 10 times larger, our method maintains nearly consistent processing quality. This indicates that our method continues to perform excellently in large-scale applications.

#### 4.2.4. Effect of Hyper-Parameters

**Figure 14:**
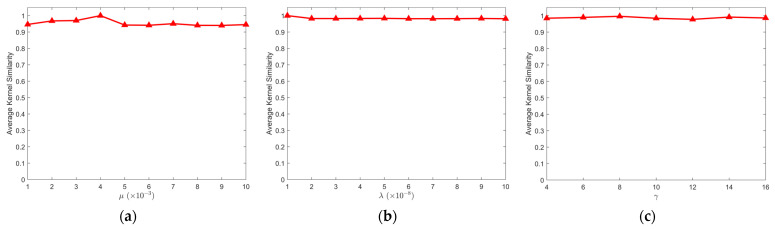
Average kernel similarity: (**a**) parameter μ; (**b**) parameter λ; (**c**) parameter γ.

Our model involves three parameters: μ, λ, and γ. A good method should be insensitive to parameter variations. Therefore, in this paper, we conduct experiments by changing one parameter at a time while keeping the other two parameters constant, and we observe the similarity of the estimated kernel to the kernel estimated by the default parameter. Figure 14 displays the average changes in kernel similarity based on the six images from Section 4.1.

Figure 14 illustrates that when we adjust the parameters to within a certain range, the kernel similarity remains stable above 0.9. Additionally, according to our observations, the results all have good visual quality. This indicates that the method proposed in this paper is stable and that the results are not significantly affected by changes in hyperparameters.

## 5. Discussion

This paper is aimed at deblurring multispectral images of the CASEarth satellite, so the inter-band gradient similarity prior is proposed. Experiments show that the method achieves visually appealing results and is not sensitive to the adjustment in hyper-parameters, which demonstrates the effectiveness and stability of the proposed method.

However, there are still some challenges to address. (1) Our approach relies on the PMP prior, which is based on natural images. Remote sensing images, however, contain more small details, so using the L0 norm may lead to the loss of some fine details. (2) CASEarth satellite MII imaging width is 300 km, resulting in large image sizes and significant data volume, making processing time critical. To improve the accuracy of kernel estimation, our method incorporates multiscale operations and numerous iterations, which increases computational time. (3) Although the joint bilateral filtering method effectively preserves the image edges, it is inherently a smoothing operation, leading to some inevitable loss of detail. There are now some methods studying the joint processing of de-striping and deblurring, but the noise in the B1 band does not exhibit the clear directional characteristics of stripe noise. Therefore, further research is needed to develop techniques for simultaneous denoising and deblurring of B1 band images.

## 6. Conclusions

In this paper, we propose a blind deblurring method for CASEarth multispectral images based on inter-band gradient similarity. The model considers the sparsity of gradient difference between bands, leading to excellent performance. Regarding the solution of the model, half-quadratic splitting and the alternating minimization method are employed. Finally, we conduct experiments on deblurring CASEarth multispectral images and compare the proposed method with five state-of-the-art methods. Both subjective and objective results demonstrate that the proposed method effectively preserves the original image’s details while enhancing edge sharpness without introducing artifacts. Additionally, the joint bilateral filtering effectively removes noise in the B1 band.

In future studies, we will focus on improving the running speed of the algorithm. Moreover, in the process of deblurring multispectral images, the preserving of spectral information is also important. Most of the existing methods are focusing on maintaining spectral information of hyperspectral images. How to apply these techniques to multispectral images is also a direction for our future work.

## Figures and Tables

**Table 1:** Parameters of CASEarth MII.

Band	Type	Wavelength (nm)	SNR (dB)	Resolution (m)	Swath Width (km)
B1	Deep blue 1	374–427	≥130	10	300
B2	Deep blue 2	410–467	≥150		
B3	Blue	457–529			
B4	Green	510–597			
B5	Red	618–696			
B6	Red edge	744–813			
B7	Near infrared	798–911			

**Table 2:** NCC values between the noisy Band1 image and other bands’ images.

Band	B2	B3	B4	B5	B6	B7
**NCC**	0.9395	0.8756	0.7944	0.7680	0.1100	0.0906

**Table 3:** Average metrics for seven bands. ** indicates significant artifacts; * indicates subtle artifacts; ^ denotes over-sharpening and distortion. Bold values represent the best metric scores, while underlined values correspond to the optimal values in subjectively good images.

Method	Figure 7			Method	Figure 8		
	P	ESL	Crms		P	ESL	Crms
Origin	0.0446	0.2870	0.5906	origin	0.0220	0.2470	0.9042
LMG *	0.0703	0.3544	0.6627	LMG **	0.0316	0.2781	0.9747
NSM **	0.0969	**0.40** **43**	**0.71** **06**	NSM **	0.0386	**0.303** **4**	0.9806
Max–min ^	**0.0** **866**	0.3817	0.8584	Max–min ^	**0.0** **396**	0.2617	**0.** **9938**
PMP	0.0676	0.3364	0.6628	PMP	0.0261	0.2539	0.9112
CPMMP	0.0692	0.3402	0.6691	CPMMP	0.0260	0.2540	0.9107
Our result	0.069 7	0.341 5	0.6716	Our result	0.02 88	0.257 6	0.91 59

**Table 4:** Average metrics for seven bands. ** indicates significant artifacts; ^ denotes over-sharpening and distortion. Bold values represent the best metric scores, while underlined values correspond to the optimal values in subjectively good images.

Method	Figure 9			Method	Figure 10		
	P	ESL	Crms		P	ESL	Crms
Origin	0.0323	0.2704	0.6096	origin	0.0402	0.2884	0.6387
LMG	0.0412	0.3006	0.6534	LMG	0.0607	0.3478	0.6926
NSM **	0.0501	0.3177	0.6551	NSM **	**0.08** **79**	**0.41** **12**	0.7532
Max–min ^	**0.0** **612**	**0.3** **301**	**0.** **7717**	Max–min ^	0.0801	0.3873	**0.** **8612**
PMP	0.0483	0.3074	0.6813	PMP	0.0607	0.3355	0.6888
CPMMP	0.0483	0.3080	0.6812	CPMMP	0.0618	0.3376	0.6913
Our result	0.05 30	0.3 120	0. 6963	Our result	0.0 697	0.3 602	0. 7602

**Table 5:** Average metrics for seven bands. ** indicates significant artifacts; ^ denotes over-sharpening and distortion. Bold values represent the best metric scores, while underlined values correspond to the optimal values in subjectively good images.

Method	Figure 11			Method	Figure 12		
	P	ESL	Crms		P	ESL	Crms
Origin	0.0210	0.2114	0.7802	origin	0.0408	0.2726	0.4775
LMG	0.0283	0.2448	0.8029	LMG	0.0678	0.3356	0.5219
NSM	0.0320	0. 2548	0.8033	NSM **	**0.09** **18**	**0.385** **5**	**0.546** **5**
Max–min ^	**0.0** **432**	**0.2** **744**	**0.** **8931**	Max–min ^	0.0868	0.3701	0.6293
PMP	0.0321	0.2445	0.8315	PMP	0.0645	0.3192	0.5294
CPMMP	0.0327	0.2466	0.8348	CPMMP	0.0642	0.3206	0.5313
Our result	0.03 49	0.2497	0. 8365	Our result	0.070 0	0.3315	0.5457

**Table 6:** Metrics for different image sizes.

Method	MII 01			MII 02			MII 03		
	P	ESL	Crms	P	ESL	Crms	P	ESL	Crms
Blurred	0.4860	0.2900	0.6548	0.0239	0.2503	0.7501	0.0401	0.2921	0.4137
400 × 400	0.0815	0.3567	0.7906	0.0336	0.2636	0.7614	0.0671	0.3464	0.5058
2000 × 2000	0.0839	0.3606	0.8093	0.0377	0.2714	0.7783	0.0662	0.3469	0.5053
4000 × 4000	0.0858	0.3653	0.8342	0.0408	0.2711	0.8178	0.0632	0.3394	0.4929

**Table 7:** Metrics for different image sizes.

Method	MII 04			MII 05			MII 06		
	P	ESL	Crms	P	ESL	Crms	P	ESL	Crms
Blurred	0.0444	0.2888	0.6262	0.0255	0.2164	0.3564	0.0484	0.2867	0.4426
400 × 400	0.0758	0.3558	0.6878	0.0419	0.2590	0.3833	0.0828	0.3489	0.5261
2000 × 2000	0.0737	0.3458	0.6761	0.0377	0.2484	0.3764	0.0821	0.3469	0.5220
4000 × 4000	0.0722	0.3445	0.6713	0.0356	0.2428	0.3725	0.0868	0.3586	0.5338

## Data Availability

The CASEarth data in this paper are free and can be downloaded from this website: https://www.sdgsat.ac.cn (accessed on 3 May 2024).
